# Virus–Host Interactions Involved in Lassa Virus Entry and Genome Replication

**DOI:** 10.3390/pathogens8010017

**Published:** 2019-01-29

**Authors:** María Eugenia Loureiro, Alejandra D’Antuono, Nora López

**Affiliations:** Centro de Virología Animal (CEVAN), CONICET-SENASA, Av Sir Alexander Fleming 1653, Martínez, Provincia de Buenos Aires B1640CSI, Argentina; adantuono@gmail.com (A.D.); noramlopar@gmail.com (N.L.)

**Keywords:** Lassa fever, arenavirus, LASV, virus–host interactions, entry, replication

## Abstract

Lassa virus (LASV) is the causative agent of Lassa fever, a human hemorrhagic disease associated with high mortality and morbidity rates, particularly prevalent in West Africa. Over the past few years, a significant amount of novel information has been provided on cellular factors that are determinant elements playing a role in arenavirus multiplication. In this review, we focus on host proteins that intersect with the initial steps of the LASV replication cycle: virus entry and genome replication. A better understanding of relevant virus–host interactions essential for sustaining these critical steps may help to identify possible targets for the rational design of novel therapeutic approaches against LASV and other arenaviruses that cause severe human disease.

## 1. Introduction

The *Arenaviridae* family includes viruses carried by mammalian hosts, classified in the Mammarenavirus genus, and members that infect reptilian hosts, which belong to the Reptarenavirus and Hartmanivirus genera [1]. Mammarenaviruses comprise 35 currently recognized species that are classified into two main groups, Old World (OW) and New World (NW) viruses. Within the NW group, viruses are divided into Clade A, Clade A-recombinant (Clade D), Clade B, and Clade C, according to their phylogenetic relationships. Clade B includes the apathogenic Tacaribe virus (TCRV), along with the known South American pathogens that produce severe hemorrhagic disease in humans: Junín virus (JUNV), the causative agent of Argentine hemorrhagic fever; and Machupo, Chapare, Guanarito, and Sabia viruses. OW mammarenaviruses include the prototypic lymphocytic choriomeningitis virus (LCMV), of worldwide distribution, and other viruses endemic to the African continent such as Mopeia (MOPV), Lujo (LUJV), and Lassa virus (LASV). LASV is the causative agent of Lassa fever (LF), a human hemorrhagic disease transmitted through contact with infected rodents (*Mastomys* spp.) that is particularly prevalent in Nigeria, Liberia, Sierra Leone, and Guinea. After infection, an average incubation time of 10 days is usually followed by general flu-like symptoms, including fever, malaise, and headache. Hemorrhagic and/or neurologic involvement can be associated with severe cases of LF [2]. Up to 500,000 infections and >5000 deaths occur every year, with mortality rates which can rise up to 50% in hospitalized patients, 90% in women in the last month of pregnancy, and nearly 100% mortality in fetuses [3]. Neurological sequelae including deafness are common features in LF survivors [4,5]. 

Arenaviruses are enveloped viruses with a negative-sense RNA genome, consisting of two single-stranded segments named S (ca. 3.4 kb) and L (ca. 7.2 kb), each encoding two proteins with an ambisense strategy for expression. The S segment encodes the nucleoprotein (NP) and the precursor of the envelope glycoprotein complex (GPC), while the L segment encodes the viral RNA-dependent RNA polymerase (L) and a matrix protein (Z) that is involved in virus assembly and budding [6]. The open reading frames, in opposite orientations, are separated by a noncoding intergenic region predicted to fold into strong stem-loop structures [7].

GPC is expressed as a single precursor polypeptide that is cleaved twice by cellular proteases to generate a stable signal peptide (SSP), a receptor-binding subunit (GP1), and a trans-membrane fusion subunit (GP2). Both the peripheral GP1 and the SSP remain noncovalently associated with GP2, and assemble into the trimeric glycoprotein (GP) complex that mediates receptor recognition and fusion of the viral and host cell membranes [8,9,10].

NP is the most abundant viral protein both in virions and infected cells, and plays critical roles during arenavirus life cycle. NP associates tightly with the viral genomic and antigenomic RNAs forming ribonucleoprotein (RNP) complexes called nucleocapsids. Nucleocapsids bind the L polymerase, constituting the biologically active units for transcription of subgenomic viral mRNAs and for viral genome replication [11,12,13]. In addition, NP interacts with the Z matrix protein and contributes to the packaging of RNPs into viral particles during virion morphogenesis [14,15,16]. Crystallographic studies revealed that LASV NP is organized in two distinct domains [17]. The N-terminal domain contains a basic crevice, initially proposed to be an m7GTP cap binding site and later reported to function in binding RNA [17,18]. The C-terminal domain of NP harbors a functional 3′-5′ exoribonuclease activity of the DExD/H-box protein family that has been shown to oppose the host type I interferon (IFN-I)-mediated immune response during viral infection. In this regard, NP is capable of degrading small viral doubled-stranded RNA fragments that could function as pathogen-associated molecular patterns, to prevent their recognition by cellular pattern recognition receptors (PRRs) [17,19,20,21]. In addition, the role of NP in the negative regulation of IFN-I production has been linked to its ability to prevent the nuclear translocation and transcriptional activity of the nuclear factor kappa B (NF-ĸB), and its direct association with the retinoic acid-inducible gene I (RIG-I) and I-kappa-B kinase epsilon (IKKε), thereby inhibiting the activation and nuclear translocation of the interferon regulatory factor 3 (IRF-3) [22,23,24].

Following arenavirus entry, nucleocapsids are delivered into the cytoplasm of the host cell where transcription and replication of viral RNA segments occur. The arenavirus Z protein directs the assembly and budding of infectious particles from the plasma membrane, co-opting proteins from the endosomal sorting complexes required for transport (ESCRT) that facilitate virus egress [25].

Over the past few years, a significant amount of novel information has accrued regarding cellular proteins that play a role in the arenavirus life cycle, including in pathogenesis, immune evasion and virus entry and egress [25,26,27,28]. Here, we summarize current knowledge on host factors that are involved in LASV entry and discuss factors crucial for LASV RNA replication. Deepening the knowledge about relevant virus-host interactions essential for sustaining these early critical steps may help identify possible targets for the rational design of novel therapeutic approaches against LASV and other arenaviruses that cause severe human disease.

## 2. Virus–Host Interactions Involved in LASV Entry

### 2.1. α-Dystroglycan (α-DG) Is the Principal Receptor for LASV Entry

Arenaviruses primarily attach to cells by binding of their surface GP to specific receptor/entry factors at the plasma membrane of host cells. α-DG was the first entry receptor discovered for LASV, as well as for other OW and for Clade C NW arenaviruses [29,30], and its interaction with GP has been widely characterized [31]. DG is expressed as a precursor and proteolytically cleaved to generate the mature α and β subunits that together serve as a molecular bridge between the extracellular matrix and the cytoplasm [32,33,34,35]. α-DG is found in the extracellular compartment, where it binds components such as laminin, and requires *O*-glycosylation to perform its biological functions [36,37], whereas the transmembrane β subunit (β-DG) docks to the cytoskeleton by associating to the cytoplasmic adaptor proteins dystrophin and utrophin [35,38,39]. DG is expressed in most cell types, but its expression patterns and glycosylation levels differ depending on the tissue [34,35].

Further reports on arenavirus biology provide evidence that the α-DG receptor requires a specific type of glycosylation for efficient virus attachment; in particular, *O*-mannosylation, rarely found in mammals [40,41]. LASV tightly binds to the “matriglycan” platform displayed on α-DG, a polymer composed of 3-xylose-α1, 3-glucuronic acid-β1 (Xylα1-3GlcAβ1-3) disaccharide repeats, which is linked to α-DG through phosphorylated *O*-mannose. Like-acetylglucosaminyltransferase (LARGE) is required for the attachment of ligand-binding moieties to phosphorylated *O*-mannose on α-DG [42]. Moreover, the recent determination of the crystal structure of the mature LASV and LCMV GPs in prefusion conformation demonstrates that the GP ectodomain engages matriglycan via multiple contacts, with a close similarity to the molecular mechanisms driving α-DG recognition of host extracellular matrix (ECM) proteins [43,44]. Therefore, it is reasonable to conceive that LASV would mimic the behavior of different host ECMs that normally interact with glycosylated membrane receptors to gain access to the cell. Indeed, post-translational modification of α-DG by the glycosyltransferase LARGE is required for both efficient LASV infection and laminin binding [45,46,47]. Mutagenesis and functional studies further identified two threonine (Thr) residues (Thr317 and Thr319) within a highly conserved amino acid motif in α-DG that play a key role in LARGE-mediated α-DG modification, and are required for recognition by LASV GP and laminin [48]. These findings further support the idea that arenaviruses displaying high affinity for α-DG may be able to compete with host ligands and displace them from the receptor to infiltrate the cell [45,48]. Thereafter, upon receptor recognition, the encounter of cellular α-DG with LASV GP induces tyrosine phosphorylation of β-DG’s cytosolic domain as well as it triggers β-DG’s dissociation from the cytoskeletal adaptor utrophin [49]. Then, it is envisioned that this later step of detachment of virus-bound DG from the actin-based cytoskeleton may ease subsequent endocytosis of the virus–receptor complex.

### 2.2. LASV Can Use Phosphatidylserine Receptors to Enter the Cell

The existence of alternative viral receptors was initially suggested by the observations that certain cell-types deficient in functional α-DG, i.e., hepatocytes, could be highly susceptible to LASV infection and that mice lacking the LARGE gene sustain LASV replication at a level comparable to that in wild-type mice [35,50]. cDNA library screening studies from Kawaoka’s group singled out the Tyro3/Axl/Mer (TAM) receptor tyrosine kinases Axl and Tyro3/Dtk as potential LASV receptor candidates [51]. TAM family members are tyrosine-kinases which expose tandem immunoglobulin-related domains to the extracellular matrix. These domains interact with host protein S (ProS) and growth arrest-specific gene 6 (Gas6), which are serum proteins that bind the negatively charged phospholipid phosphatidylserine (PtdSer). PtdSer is translocated from the inner leaflet to the external leaflet of the plasma membrane in apoptotic cells, where it acts as a signal for professional phagocytes (macrophages and dendritic cells) as well as non-professional phagocytes (e.g., epithelial cells) [52,53]. TAM receptors have been shown to play a role in virus entry of several RNA viruses such as Ebola (EBOV), dengue (DENV) and Zika, through a mechanism termed “apoptotic mimicry” [54,55,56]. This mechanism involves recognition of PtdSer exposed on the viral surface, incorporated from the cellular lipid bilayer during the budding process, as a signal for virus uptake [57,58,59]. Of note, there is evidence that cells infected with the NW arenavirus Pichinde display PtdSer on their plasma membranes; therefore, it is reasonable to conceive that both NW and OW arenaviruses could benefit from PtdSer receptors for viral entry [60]. In the case of LASV, initial studies using a HIV-based lentiviral vector pseudotyped with LASV GP empirically confirmed the capability of Axl to facilitate viral entry to cells lacking optimal carbohydrate modification of α-DG or to DG knockout cells [51]. Experiments from the Choe’s group applying alternative lentiviral pseudotyped platforms showed no enhancement of LASV (or LCMV) entry upon overexpression of Axl in human embryonic kidney (HEK-293T) cells [61] and hypothesized that LASV internalization via α-DG may be preferred over PtdSer receptors. Later, experiments from the Kunz’s group using a recombinant LCMV system expressing LASV GP (rLCMV-LASVGP) ultimately confirmed that the endogenous expression of Axl does not actually enhance viral entry in the presence of fully functional α-DG receptor but it strongly augments viral infection in the absence of α-DG [62].

In line with these findings, T-cell immunoglobulin mucin I (TIM-1) has also been recently identified as a PtdSer receptor for LASV entry [63]. TIM receptors are cell surface glycoproteins that display an extracellular immunoglobulin variable-like domain (IgV), bearing a structural pocket with high affinity for PtdSer [64]. Unlike TAM receptors, TIM directly binds PtdSer, without the need for the Gas6 or ProS adaptors. It was demonstrated that TIM-I also mediates entry of vesicular stomatitis virus pseudovirions bearing LASV GP, either in the absence of α-DG or under conditions where it is inadequately glycosylated [63]. This behavior resembles that of Axl, suggesting a similarity between the entry route promoted by TAM and TIM receptors. In this sense, although functional α-DG would be the first LASV receptor of choice, the use of PtdSer receptors could function as a non-canonical GP-independent mechanism exploited by the virus to expand the spectrum of cellular tropism.

### 2.3. DC-SIGN and LSECtin Lectin Receptors Can Mediate LASV Cell Entry

**Figure 1:**
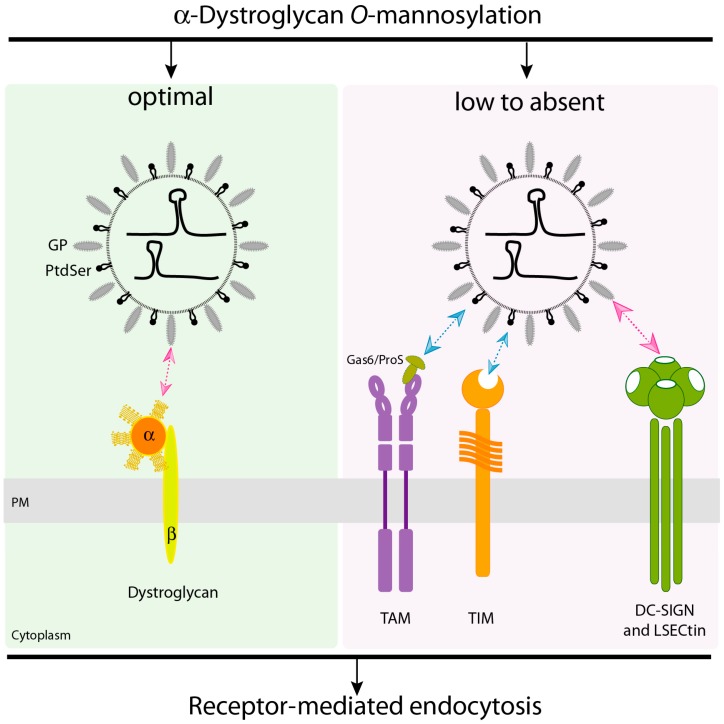
Model of cell receptor/s recognition by Lassa virus (LASV). Left panel. The α-dystroglycan (α-DG) receptor needs to be *O*-mannosylated for efficient virus attachment. In the presence of a fully functional α-DG receptor, LASV enters host cells after binding to the matriglycan platform displayed on α-DG. Right panel. In the absence of α-DG or in conditions where it is inadequately glycosylated, phosphatidylserine (PtdSer)-binding receptors (TAM; TIM) and C-type lectin receptors (DC-SIGN; LSECtin) can mediate α-DG-independent entry. TAM kinases bind Gas6 or ProS serum proteins, which bind to PtdSer molecules exposed on the viral envelope membrane. TIM directly binds PtdSer, without a need for the Gas6 or ProS adaptors. C-type lectins interact with glycans on the LASV glycoprotein (GP). PM: plasma membrane; TAM: Tyro3/Axl/Mer; TIM: T-cell immunoglobulin mucin; DC-SIGN: dendritic cell-specific intercellular adhesion molecule-3 nonintegrin; LSECtin: liver and lymph node sinusoidal endothelial calcium-dependent lectin. Cartoon diagram not to scale.

Two additional receptor candidates were identified in the cDNA screening studies [51]: dendritic cell-specific intercellular adhesion molecule -3 grabbing nonintegrin (DC-SIGN) and liver and lymph node sinusoidal endothelial calcium-dependent lectin (LSECtin), both of which belong to the calcium-dependent (C-type) family of lectins. Of note, there is previous evidence indicating that DC-SIGN can facilitate infection of the NW JUNV and other enveloped viruses, including EBOV and Rift Valley fever virus [65,66,67]. Likewise, LSECtin has been implicated in entry of EBOV, SARs coronavirus and Japanese encephalitis virus [68,69,70]. As observed for TAM receptors, both DC-SIGN and LSECtin enhanced the susceptibility of cells to infection by LASV GP-pseudotyped lentivirus and participate in LASV entry independently of α-DG [51]. These lectin receptors were more effective at enhancing virus infection than TAM receptors, but importantly, none of these alternative receptors showed higher efficiency than properly modified α-DG, the principal portal of entry for LASV [51] (Figure 1). It was also shown that DC-SIGN or LSECtin binding to LASV GP is carbohydrate-specific, a fact that is characteristic of this C-type lectins family [51]. Strikingly, experiments using monocyte-derived immature human dendritic cells (MDDCs), which lack expression of Axl and Tyro3, demonstrated that upregulated expression of DC-SIGN correlates with enhanced virus attachment and productive infection, and that highly mannosylated glycans exposed on LASV GP1 surface interact with DC-SIGN during attachment [71]. Thus, DC-SIGN and LSECtin may facilitate LASV entry into dendritic cells, which represent the preferred early targets for arenavirus infection [72,73].

### 2.4. LASV Entry Involves Macropinocytosis and Intracellular LAMP1 Receptor for Virus Fusion

Upon receptor binding, OW arenaviruses including LASV, enter the host cell by a clathrin-independent endocytic process followed by transport to late endosomal compartments, where pH-dependent fusion of viral and cell membrane takes place [74,75]. Strikingly, sodium hydrogen exchangers (NHEs) have been identified through a genome-wide small interfering RNA screen, as host factors involved in the multiplication of LCMV in human cells [76]. Based on pharmacological and genetic analysis, Iwasaki et al. further validated NHE as entry factors for LCMV and LASV, implicating macropinocytosis in arenavirus entry [77]. Moreover, using the pseudotyped rLCMV-LASVGP and a panel of specific inhibitors for cellular factors involved in the regulation of macropinocytosis, Oppliger et al. showed that DG-mediated LASV entry depends on regulatory factors, including NHE, associated with this pathway [78]. 

An unbiased haploid genetic screening in α-DG-deficient cells pinpointed the lysosome-associated membrane protein 1 (LAMP1) as a late endosomal co-receptor specifically required for efficient LASV entry [79]. LAMP1 is mostly found in lysosomes, but it also locates in other endosomal structures. It is hypothesized that the acidic pH of the late endosome destabilizes the high affinity interaction between LASV GP and α-DG, resulting in a “receptor switch” to LAMP1. In this sense, LAMP1 would work as a secondary intracellular receptor that helps induce the GP conformational changes needed for virus fusion. This interpretation is supported by a series of biochemical studies that demonstrated that LAMP1 directly interacts with LASV GP in a pre-fusion configuration, and which described that the strength of this interaction is modulated by pH conditions, where a drop in pH can destabilize LASV GP affinity for α-DG, thereby inducing potent binding to LAMP1 [79]. Furthermore, structural and functional studies showed that LASV GP1 conformation is stable at low pH conditions, at which it displays a triad of histidine residues that are involved in LAMP1 binding [80,81]. In this regard, LAMP1 facilitates LASV exit from earlier endosomal compartments, avoiding prolonged exposure to a harsh proteolytic environment, increasing the overall efficiency of LASV entry and infection [82]. Besides LAMP1, the haploid genetics screening also pointed out α-2,3-sialyltransferase ST3GAL4, as well as additional factors involved in N-glycosylation and sialylation, as being important for LASV entry. Mutations in ST3GAL4, yielding a specific deficiency in sialylation of LAMP1, totally abrogate its ability to interact with LASV GP [79]. This highlights the strict requirement of a specific glycosylated version of LAMP1 for the biochemical interaction with GP to take place. In sum, it is envisioned that LASV would initially attach to the cell surface via α-DG, a step which would lead to delivery of virions to endosomes. Later, as virus-containing vesicles acidify, LASV would dissociate from the α-DG receptor, gaining affinity for LAMP1, and therefore completing the internalization process in a LASV-unique manner that is distinguishable from a standard endocytic process [83,84].

Altogether, these findings describing virus–host interactions involved in LASV entry disclose the diverse spectrum of mechanisms implemented by the virus to efficiently fulfill its internalization and fusion. On the one hand, the ability to utilize entry strategies alternative to α-DG receptor (such as PtdSer or lectin receptors), provides the virion with versatility to enlarge its cell tropism and promotes access to selected cell-targets such as dendritic cells, which are high privileged sites for early LASV productive infection. On the other side, LASV engagement of the late endosomal receptor LAMP1 likely guarantees the optimal spatial conditions required for virus fusion in close proximity to the endosome membrane. Given that not only α-DG- but also Axl-mediated entry involve LAMP1 co-factor [62], it is conceivable that multiple pathways converge at similar late endosomal compartments to efficiently accomplish LASV entry.

## 3. Role of Virus–Host Interactions Involving the LASV Replication Complex

### 3.1. Role of DEAD-Box RNA Helicase 3 (DDX3) in Viral Replication

A series of large-scale proteomic studies applying mass-spectrometry have been undertaken to comprehensively identify novel human protein candidates that could interact with the arenavirus proteins [85,86,87,88,89]. Special attention has been paid to NP-binding partners due to the multifunctional role of NP in the viral cycle, which involves crucial interactions with L and Z viral proteins [12,13,15,16,17,90], and its ability to hijack host factors to inhibit the antiviral innate immune response [19,22,24,91]. It is believed that identifying essential NP–host cell protein interactions can pave the way in the rational design of novel strategies to tackle arenavirus infections. One of the LASV NP interactors recently identified in human cells is DDX3, a protein belonging to the DEAD (Asp-Glu-Ala-Asp) box RNA helicase family, which harbors ATPase and RNA helicase activities [89]. Of note, DDX3 has also emerged in proteomic studies of virus-infected cells, as a novel interacting partner of the OW LCMV and the NW JUNV NPs [85]. CRISPR/Cas9-mediated deletion of DDX3 gene has been shown to lead to a significant reduction in virus yields of LASV, LCMV or JUNV in cell culture. Subsequently, lentiviral-mediated reconstitution of DDX3 expression resulted in a notable recovery in the infection rate of the three viruses, indicating a relevant role of DDX3 in virus growth as a proviral cellular factor [89]. 

DDX3 is known to be involved in multiple steps of RNA metabolism, including RNA transcription and the initiation of translation in host cells [92,93,94]. As other DEAD-box RNA helicases, such as DDX1 and DDX5, DDX3 appears to facilitate replication of different RNA viruses, as the alphavirus Venezuelan equine encephalitis virus and the hepatitis C virus (HCV), among others [95,96,97,98,99,100]. DDX3 is also required for translation of mRNAs containing a long or structured 5′ untranslated region (UTR), such as human immunodeficiency virus type-1 (HIV-1) genomic RNA (gRNA). Indeed, it was reported that DDX3 interacts with the 5′ region of the target mRNA, binds the eukaryotic translation initiation factor 4G (eIF4G) and poly A-binding protein cytoplasmic 1 (PABP), and interacts with HIV-1 Tat protein to facilitate translation of HIV-1 mRNAs [96,101]. In reference to arenaviruses, it was demonstrated that translation of a synthetic arenavirus mRNA analog was unaffected in DDX3-deficient cells, indicating no critical engagement of DDX3 in viral mRNA translation initiation [89]. In contrast, minireplicon assay-based experiments demonstrated that the pro-arenaviral activity of DDX3 strongly depends on DDX3’s ability to promote viral RNA synthesis, involving both previously described DDX3 ATPase and helicase RNA-unwinding activities in this function [89,102].

Strikingly, alternative roles have been ascribed to DDX3 in the context of different viral infections [103,104]. On the one hand, DDX3 is considered an antiviral factor given that it is involved in the innate immune response against some viruses such as HIV-1, DENV and HCV [105,106,107]. DDX3 has been shown to collaborate in the production of IFN-I, through interaction with components of the RIG-I-mediated IFN-I induction pathway [108,109,110]. However, in contrast to this IFN-I promoting capacity of DDX3, mechanistic analysis has provided evidence that, in the case of LCMV, DDX3 suppresses the IFN-I response at late times of infection, still it remains to be confirmed whether this IFN-I-suppressive role of DDX3 is sustained in the context of an infection with the pathogenic LASV [89]. Secondly, DDX3 is known to be an essential component for stress granule (SG) assembly, and to interact with other SG proteins, such as eIF4E [111]. Different proteomic approaches based on mass-spectrometry have singled out new arenavirus NP-binding candidates related to the SG biology; including but not limited to the Ras GTPase-activating protein-binding protein 1 (G3BP1), eIF2α, apoptosis-inducing factor mitochondrion-associated 1 (AIFM1) and PABP, yet none of them have been confirmed as LASV interactors by alternative biochemical methods [85,89]. Of note, colocalization experiments have revealed the association of the NW arenavirus TCRV replication–transcription complexes (RTCs), where NP accumulates, with G3BP1 and a non-canonical collection of ribosomal proteins, including the ribosomal proteins RPS6 and RPL10a, as well as translation initiation factors eIF4G and eIF4A [112]. In this regard, the finding that JUNV infection inhibits SG formation [113] might be related to the NP-mediated sequestration of DDX3 and other SG-related proteins, resulting in the lack of availability of essential factors needed for SG nucleation. Similarly, in the case of influenza virus infections, it has been hypothesized that the interaction of DDX3 with the viral NS1 protein prevents DDX3 binding to eIF4E and PABP1 as well as DDX3–NP interaction, thus suppressing SG formation, NP recruitment into SGs, and DDX3 antiviral activity [114]. Therefore, it is possible that in a similar way, LASV NP may counteract DDX3 antiviral function and in turn use DDX3 to enhance its own replication.

### 3.2. Other RNA Helicases Potentially Involved in LASV Replication

**Figure 2:**
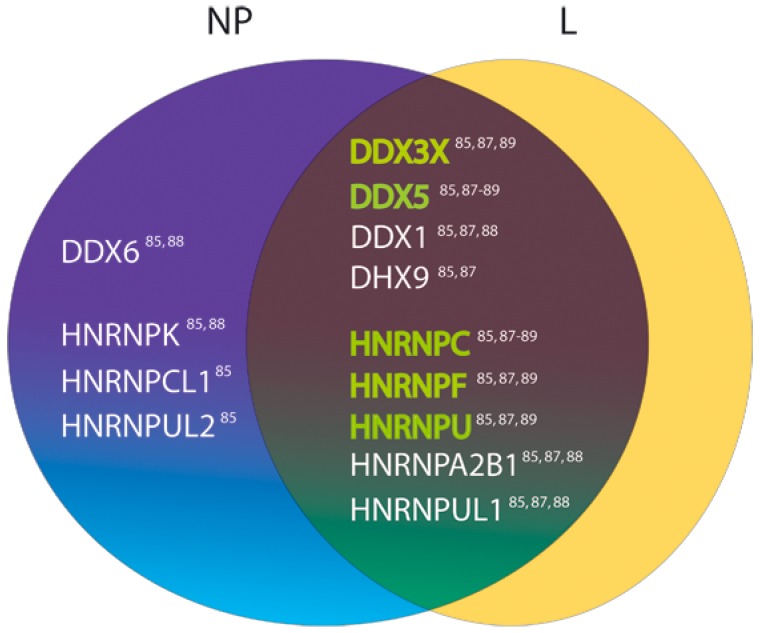
Cellular DExD/H-box helicases and heterogeneous nuclear ribonucleoproteins (hnRNPs) identified among binding partners of the nucleoproteins (NPs) of LASV, lymphocytic choriomeningitis virus (LCMV), and/or Junín virus (JUNV) in different proteomics approaches [85,88,89]. Targets common to the LCMV L polymerase [87] are depicted. LASV NP binding partners are highlighted in green. References are indicated for each target.

In addition to DDX3, a number of cellular proteins functionally related to RNA biosynthesis and ribonucleoprotein complex assembly, including the DEAD-box helicase 5 (DDX5, also referred to as RNA helicase p68) and RNA helicase A (namely DHX9), members of the DExD/Hbox protein family, have been identified as potential overlapping targets of the LCMV L protein and the NP of LASV, LCMV, and/or JUNV (Figure 2) [85,87,89]. Moreover, they have already been confirmed as interactors of the RNA-dependent RNA polymerase (RdRp) of other RNA viruses. For example, DDX5 has been shown to interact with the C-terminal region of HCV NS5B, and has been suggested to be part of the HCV replicase complex [115]. Similarly, DDX5 associates with the influenza A virus PB1 and PB2 proteins [116] and it is needed for an efficient activity of the viral polymerase [117]. Evidence has been provided that DHX9 interacts with the viral genomic RNA and non-structural protein 3 (nsP3) within active replication complexes in Chikungunya virus (CHIKV)-infected cells, displaying an inhibitory effect on viral RNA synthesis and an enhancing effect on viral genome translation, which may imply a regulatory role in CHIKV life cycle [118]. Porcine reproductive and respiratory syndrome virus (family *Arteriviridae*) nucleocapsid protein interacts with DHX9 polymerase to overcome premature termination of viral RNA synthesis [119]. Overall, DExD/H-box helicases emerge as host factors selectively hijacked by polymerases and nucleocapsid or nonstructural proteins from several viruses to facilitate their multiplication. Further work must be carried out to validate the binding of DDX5 and DHX9 helicases to LASV and/or other arenavirus proteins and provide a mechanistic model that could explain the relevance of these interactions.

Given the observation that many host factors converge as common binding-partners of RNA viruses, it is intriguing whether this is a consequence of the conserved conformational structure shared among proteins from different families of viruses. In particular, segmented negative strand viruses (sNSV) polymerases share key conserved motifs, specifically those corresponding to the fingers, palm and thumb subdomains within the RdRp domain located in the central part of the polypeptide chain. The central ring-like RdRp domain is linked to appendages that would be dedicated to 5′ mRNA capping activities [120,121,122,123]. Based on the amino acid sequence motif conservation displayed by sNSV polymerases and the structural similarity between the polymerases of the bunyavirus La Crosse encephalitis virus and influenza virus revealed by crystallographic studies, an overall structural configuration has been proposed for arenavirus L protein. It consists of a canonical RdRp core with N- and C- extensions that form a cavity connected to the exterior by four tunnels (NTP entry, template entry, template exit, and product exit) [124,125]. Structural information has led to a model for sNSV vRNA synthesis in which the L protein would operate in either transcription mode or replication mode, not only during initiation, but also along the whole RNA synthesis process [125,126]. A key question that needs to be addressed is how the polymerase switches from one mode to the other. For arenaviruses, there is genetic and biochemical evidence that L–L interaction is essential for polymerase activity, suggesting that the L polymerase may function in an oligomeric conformation [127]. Thus, as proposed for influenza virus [128,129], a conformational transition of the polymerase from a monomeric state during transcription to an oligomeric state during replication might be hypothesized. In this sense, it is probable that the association with cellular partners may additionally modulate the switch from the transcriptase to the replicase mode of the arenavirus polymerase. To date, DDX3 is the sole NP and L protein interactor for LASV or any other arenavirus that has been demonstrated to contribute in viral RNA synthesis, although the precise underlying mechanism remains to be fully understood. However, it is tempting to speculate that the binding of DDX3 (and eventually other cellular RNA helicases) with NP and L in a replicase complex, may contribute to the unwinding of viral RNA secondary structures such as RNA hairpins within the non-coding intergenic regions, which must be read-through during replication to accomplish full length genome and antigenome RNA synthesis. Likewise, DDX3 and/or other host helicases may facilitate encapsidation of the nascent chain by NP during replication.

### 3.3. Host Heterogeneous Nuclear Ribonucleoproteins as Candidate Factors Required for the LASV Life Cycle

Other host factors that have recently emerged as potential candidate partners of LASV as well as LCMV and JUNV NP and/or L polymerase are the components of the heterogeneous nuclear ribonucleoprotein (hnRNP) family, including hnRNPA2/B1, one of the most abundant hnRNPs belonging to the A/B type [85,88,89]. HnRNPs are RNA-binding proteins involved in processing pre-mRNAs as well as in mRNA translation, trafficking and stability. Notably, previous reports have already demonstrated the interaction between JUNV NP and hnRNP A1 and that depletion of hnRNP A1 and A2 caused a strong inhibition of virus yield, suggesting a key role of hnRNPs A/B in JUNV multiplication [130]. Additionally, hnRNP K, an LCMV and JUNV NP binding partner detected in the proteomic screening by King et al. [85], has also been reported as a necessary host factor required for JUNV multiplication [131]. Likewise, studies from different groups have similarly ascribed relevant functions to hnRNPs in other viral infections. For example, hnRNP A2/B1 has been proposed to work as a positive regulator in viral RNA synthesis of influenza A virus, hnRNP A2 has been shown to regulate the trafficking of HIV-1 genomic RNA, and hnRNP K has been shown to support vesicular stomatitis virus replication by regulating cell survival and cellular gene expression [132,133,134]. Altogether, although further research needs to be completed to unveil the importance of hnRNPs in the *Arenaviridae* family, it is intriguing whether any of these proteins would specifically associate with the LASV RNP complex, playing a critical function either in viral genome transcription and/or replication.

### 3.4. Z Protein Interactors

Arenavirus Z matrix protein has been proposed to drive a mechanism to ensure accurate packaging of all necessary virion components. Apart from its pivotal role in virus assembly and budding [135,136], Z also inhibits viral RNA synthesis by directly binding the L polymerase to trigger its catalytic inactivation, and this can still occur in the absence of host cellular factors [137,138]. Of note, it has been hypothesized that the Z–L complex not only guarantees downregulation of viral gene expression, but also serves as a platform for the functional polymerase to remain locked on the template and be properly packaged into the mature virion [138]. Interestingly, a recent proteomics approach based on JUNV Z has pinpointed a number of targets that were incorporated to Z virus-like particles (VLPs) and purified JUNV particles and which include common interactors of the L and NP proteins, such as DDX3, DDX5, DHX9, hnRNPA2/B1, and PABP [86]. Actually, this is not a totally unexpected observation. For instance, it has been demonstrated for HIV-1 that DHX9 protein stimulates transcription of HIV-1 RNA as well as it associates with the Gag protein to ensure it is adequately recruited into virus particles during the assembly process [139]. In line with this, it is predictable that the association of Z protein with key cellular factors required for viral RNA synthesis may concomitantly facilitate virus packaging and/or make these factors available to complement the activity of viral RNP upon cell entry.

## 4. Concluding Remarks

LASV is currently considered a top priority emerging pathogen causing severe hemorrhagic fever outbreaks [140]. At this moment, treatment is limited to the nonspecific antiviral ribavirin, shown to be partially effective in LASV infections [141], and the use of favipiravir is still under investigation [142,143]. Moreover, there is no FDA-approved vaccine against LASV or any other arenavirus to date. Therefore, there is an urgent need to develop novel approaches to combat and prevent the infection with these viruses.

In the last years, several reports have provided robust evidence that LASV can utilize alternative entry factors apart from the well-characterized α-DG [144]. Novel lectin and PtdSer receptors have emerged as non-canonical LASV ports of entry, proposed to enhance the viral cell tropism and/or redirect infection to selected cell types.

Studies on virus–host interactions have also improved our understanding of the mechanisms driving LASV replication. A recent series of proteomic approaches oriented to the analysis of the interactome of arenavirus NP, L, and Z proteins in human cells singled out several host factors, such as DExD/H-box helicases and heterogeneous nuclear RNPs, which might be potentially involved in arenavirus RNA synthesis. Particularly, it was demonstrated that the DDX3 ATP-dependent RNA helicase is a LASV target and may be dually exploited to both suppress the host immunity and promote viral replication and/or transcription, since DDX3 ATPase and helicase activities are involved in promoting optimal levels of viral RNA synthesis. Notably, it has recently been reported that a chemical compound directed to an RNA binding site of DDX3 protein displayed broad-spectrum antiviral activity against HIV drug-resistant strains, HCV, DENV, and West Nile virus infection [145]. In this regard, it is intriguing whether the use of DDX3-blocking compounds could be tentatively applied as a novel weapon to battle LASV infections.
